# Prioritizing Health: A Systematic Approach to Scoping Determinants in Health Impact Assessment

**DOI:** 10.3389/fpubh.2016.00170

**Published:** 2016-08-22

**Authors:** Lindsay C. McCallum, Christopher A. Ollson, Ingrid L. Stefanovic

**Affiliations:** ^1^Department of Physical and Environmental Sciences, University of Toronto, Scarborough, ON, Canada; ^2^Intrinsik Environmental Sciences Inc., Mississauga, ON, Canada; ^3^Ollson Environmental Health Management, Ancaster, ON, Canada; ^4^Faculty of Environment, Simon Fraser University, Burnaby, BC, Canada

**Keywords:** health impact assessment, healthy public policy, public health, scoping, health determinants, impact assessment

## Abstract

The determinants of health are those factors that have the potential to affect health, either positively or negatively, and include a range of personal, social, economic, and environmental factors. In the practice of health impact assessment (HIA), the stage at which the determinants of health are considered for inclusion is during the scoping step. The scoping step is intended to identify how the HIA will be carried out and to set the boundaries (e.g., temporal and geographical) for the assessment. There are several factors that can help to inform the scoping process, many of which are considered in existing HIA tools and guidance; however, a systematic method of prioritizing determinants was found to be lacking. In order to analyze existing HIA scoping tools that are available, a systematic literature review was conducted, including both primary and gray literature. A total of 10 HIA scoping tools met the inclusion/exclusion criteria and were carried forward for comparative analysis. The analysis focused on minimum elements and practice standards of HIA scoping that have been established in the field. The analysis determined that existing approaches lack a clear, systematic method of prioritization of health determinants for inclusion in HIA. This finding led to the development of a Systematic HIA Scoping tool that addressed this gap. The decision matrix tool uses factors, such as impact, public concern, and data availability, to prioritize health determinants. Additionally, the tool allows for identification of data gaps and provides a transparent method for budget allocation and assessment planning. In order to increase efficiency and improve utility, the tool was programed into Microsoft Excel. Future work in the area of HIA methodology development is vital to the ongoing success of the practice and utilization of HIA as a reliable decision-making tool.

## Introduction

The determinants of health are those factors that have the potential to affect health, either positively or negatively, and include the range of personal, social, economic, and environmental factors (1). Some of these factors are related to the aspects of biological or genetic makeup that cannot be changed, while others are the result of personal circumstances (i.e., lifestyle choices, employment, income, etc.). Further, both individual- and population-level health have the potential to be affected by changes in the environment, including social, built, and natural environments. These changes are often not directly linked to health or health care but impact health *via* indirect pathways (2).

In the practice of health impact assessment (HIA), the stage at which the determinants of health are considered for inclusion in the evaluation is during the scoping step. The scoping step is intended to identify how the HIA appraisal will be carried out and to set the boundaries (e.g., temporal and geographical) for the assessment (3). This is also typically the first stage where stakeholders are able to provide input into the HIA process. Since scoping is intended to set up a blueprint for the entire HIA, it is a vital part of the process that continues to guide and focus the practice going forward (4, 5).

There is an established set of “Minimum Elements and Practice Standards” that have been widely employed in the HIA field and provide guidance on conducting scoping (6). The guidance specifies that a range of health issues to be examined in the HIA should be identified, specifically, that scoping should include systematic consideration of potential pathways (direct, indirect, and cumulative), and the final scope should focus on those impacts with the greatest potential significance when factors, such as impact, stakeholder priorities, and equity, are taken into consideration. However, one issue with the scoping step of HIA is that there is currently no consistent and transparent way of identifying priorities when it comes to assessing determinants. Rather, it is typically a subjective determination made by those conducting the scoping exercise. This can pose a problem when complex projects have a multitude of determinants that could impact heath and should be included in the HIA. This problem becomes even more apparent when there is a limited amount of funding for the assessment.

**Figure 1:**
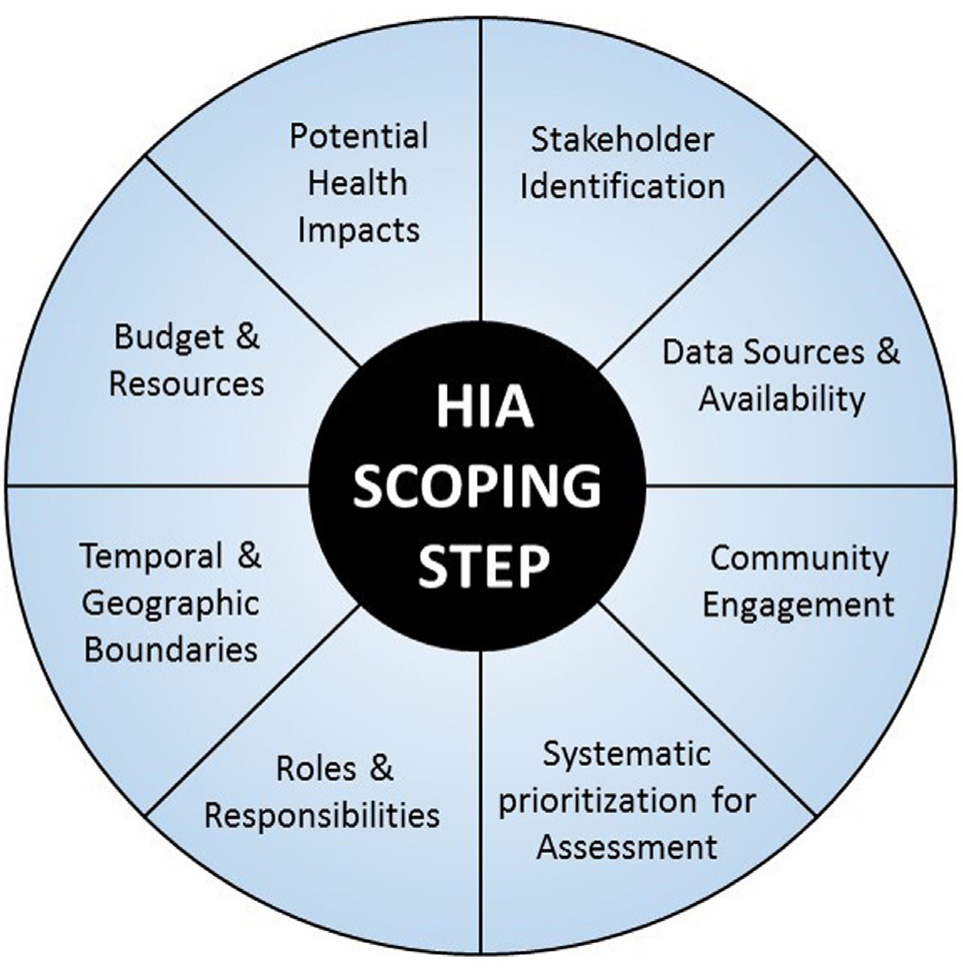
**Key factors involved in HIA scoping step (6)**.

There are several factors that can help to inform the scoping process, many of which are considered in existing HIA tools and guidance (Figure 1); however, a clear, transparent, and systematic method of prioritizing determinants is lacking. The objective of this research was to evaluate the current inventory of HIA scoping tools to identify their strengths and deficiencies, focusing on prioritization of determinants and systematic methodologies for scoping. Based on these findings, a tool was developed to enhance the practice of HIA scoping by providing a systematic method of prioritizing health determinants for inclusion in the assessment while including consideration of data gaps and budget constraints. This tool will improve upon currently employed practices around HIA scoping that are often discretionary and can lack sufficient transparency or consistency.

## Materials and Methods

In order to analyze the existing HIA scoping tools that are available, a systematic literature review was conducted in the spirit of the Cochrane Handbook for Systematic Reviews, which is based on the notion that science is cumulative, and by taking a weight-of-evidence approach, decisions can be made based on the best information available (7). The search included both the primary peer-reviewed literature and the publically available gray literature, which were both screened based on a specific set of inclusion criteria. These included (i) articles published in English; (ii) must be related to formal HIA rather than other forms of impact assessment (i.e., risk assessment, environmental assessment, equity assessment, socioeconomic assessment, etc.); (iii) clearly identified as a HIA tool, with a methodology or process to follow (i.e., toolkits, workbooks, worksheets, grids, checklists, etc.) rather than general HIA guidance documents; and (iv) applicability across a range of scenarios and sectors rather than specific HIA case studies.

The primary literature search strategy included using the search terms “health impact assessment AND scoping” to seek out relevant articles in several interdisciplinary databases including: Scopus, Web of Science, and PubMed. This search resulted in a total of 96 articles with duplicates omitted. A Tier I screening included a review of titles and abstracts, which reduced the total results to 23 primary articles. This was followed by a Tier II screening, which included a full-text review to determine relevancy, based on the inclusion/exclusion criteria applied, resulting in one primary article being carried forward for comparative analysis.

The gray literature search was conducted using the Google internet search engine. In order to maintain consistency, the same search terms were applied, producing 45,100 results. The first 500 (as they appear; in order of relevance) were screened. A Tier I screening was completed looking only at the source title and description, resulting in 92 potentially relevant resources with duplicates omitted. A Tier II screening was then conducted, which included a preliminary full-text review. Often, this consisted of reviewing an executive summary, introductory chapter, or results section, to determine whether the resource met the inclusion/exclusion criteria. This was considered necessary at this stage as some of the documents were hundreds of pages in length. This Tier II screening resulted in nine resources being carried forward for full-text review and comparative analysis.

**Figure 2:**
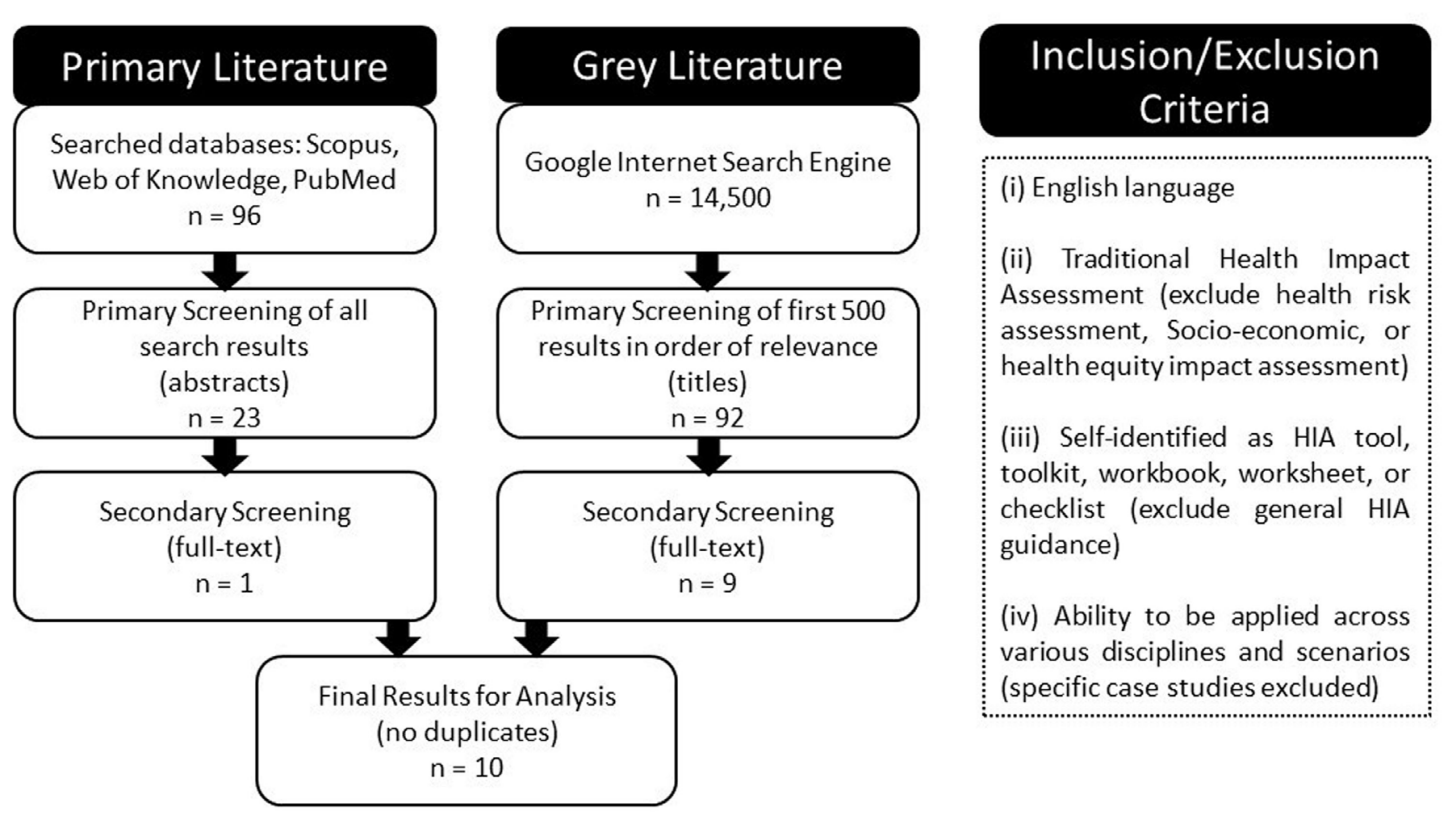
**Literature review strategy and inclusion criteria for HIA scoping tools**.

In total, the literature search produced 10 distinct HIA scoping tools that met the inclusion/exclusion criteria, all of which were included in a comparative analysis to identify any gaps in current methodology and inform development of more robust HIA scoping methods and processes (Figure 2).

Based on the comparative analysis, a method development process was undertaken to address gaps identified in existing HIA scoping practice. This was an iterative process that involved development of a specific methodological approach and theory, followed by review and revision, development of a user interface, internal testing and final revision. The results of both the comparative analysis and resulting methodology are provided in detail below.

## Results

The results of the literature search culminated in a comparative analysis of existing HIA scoping tools in order to identify the level of transparency and consistency associated with current methodologies, particularly regarding the selection of health determinants for inclusion in HIA.

### Analysis of Existing HIA Tools

The results of the literature search indicate that the majority of HIA tools are part of the publically accessible gray literature, with only one tool published in the primary literature. In total, 10 HIA scoping tools were identified that met the inclusion/exclusion criteria. All of these tools were then analyzed to determine whether they met certain criteria, particularly with respect to scoping of health determinants. These criteria were adapted from the Minimum Elements and Practice Standards, which have been developed and widely used in the field of HIA (6). Although these standards “are not rigid criteria for acceptability but represent the authors’ perspective on best practices,” they have been extensively applied as a method of quality assurance among practitioners and reviewers alike. The results of the comparative analysis are provided in Table 1.

**Table 1:** **Comparative analysis of existing HIA scoping tools**.

HIA scoping tool	General approach	Stakeholder engagement approach?	Provides guidance on determinant selection?	Establishes a plan for carrying out HIA?	Systematic method of focusing on impacts of greatest significance?	Priority health issues identified through key factors?	Establishes roles and responsibilities for HIA team?	Budget considerations?
Bert et al. (8)	Scoping grid to characterize impacts	No	Yes: provides a list of 33 potential impact areas	No	Limited: impacts characterized using a 5-point scale [highly negative (−2) to highly positive (+2)]	Limited: impact and identification of higher risk groups characterized	No	No
Castonguay and St-Pierre (9)	Series of key questions	No	No: but suggests creating a “logic model”	Limited: identifies type of HIA (rapid, intermediate, comprehensive) and parameters	No	No	Limited: asks for team identification (project management role, scientific role, knowledge brokering role)	Limited: asks to identify parameters (temporal, geographic, financial)
CREW (10)	Toolkit: key questions, tips, and general information	Yes: suggests involvement and provides some examples of typical stakeholders and engagement tips	Yes: provides a table of 47 possible health determinants	Limited: asks key questions and considers sources of evidence	No	Limited: impacts generally characterized, but no clear priority determination	Limited: asks question “roles and responsibilities?”	Limited: asks question “what financial and human resources are available?”
DDL (11)	Scoping worksheet	Limited: “stakeholders” and “community engagement” are listed as key details to be filled out	Limited: provides space for a causal pathway to be developed	Yes: scoping tables include input for details of assessment, evidence/data, boundaries, deadlines, and reporting requirements	Limited: table provided to identify prioritized health impacts, but no systematic methods	Limited: identifies the need for priority health issues to be identified, but no basis other than causal pathway	Yes: an entire table is dedicated to details on HIA activities, timelines, who is responsible, and who will review	No
HIP (12)	Scoping exercise and worksheet	Limited: suggests various outreach options to solicit feedback	Yes: provides example pathway diagrams to help identify health outcomes	Limited: worksheet allows for input of data sources and methods to be used	Limited: worksheet allows for priority input, but no systematic approach	Limited: worksheet allows input of key information and priority, but no clear link	Limited: identifies it as an essential task, but not included in the worksheet	No
IPH (13)	Scoping table provides key questions and general info	Yes: requires identification of steering committee; stakeholders, informants	No	Yes: requires details on evaluation of evidence, reporting information, recommendations, and evaluation protocols	No	No	Yes: provides a breakdown of key requirements and tasks and asks for assignment of responsibility for each	Yes: asks about costs and identifies key areas for budgetary consideration
Metro Vancouver (14)	Series of scoping tools	Limited: provides stakeholder assessment table to fill out with key contacts and level of importance	Limited: suggests creation of an “activity impact influence diagram” providing limited health determinant examples	Yes: provides a detailed section to identify the HIA terms of reference and a scoping checklist to identify the type of HIA	Limited: determination of priority is based on user judgment of several factors	Yes: uses impact and likelihood as factors to determine priority	Yes: requires comprehensive terms of reference to be signed off, includes roles and responsibilities of team members	No
PHAC (15)	Key questions and scoping checklist	Limited: asks about community concerns and identification of key stakeholders	No	Limited: provides a scoping checklist to determine level of HIA (“brief vs. more thorough”) and asks key questions about data	No	No	Limited: asks who will conduct HIA and skills needed	Limited: asks about “budget and sources of funding”
Vohra et al. (16)	Scoping table with key question/issues *(only Section 3 included in analysis)*	Limited: asks to identify which professional and community stakeholders will be consulted	Limited: asks whether there are any specific health impacts the HIA should focus on	Limited: asks questions about what approach/model will be used for the HIA, study population, and geographic area	No	No	Limited: asks questions regarding who is responsible for project management and who is on the steering committee	No: no questions about budget in the scoping section of the toolkit
WHAISU (17)	Scoping checklist with guidance notes	Limited: asks “who are the stakeholders?” and states that their involvement is important	No	Limited: asks questions about HIA type, boundaries, methods	No	Limited: suggests focusing on impacts most likely to occur and affect health	Limited: asks to identify roles and responsibilities	Limited: asks “what financial and human resources are available?”

Based on the analysis conducted, it is apparent that there is a wide range of approaches and tool formats for HIA scoping. Out of the tools reviewed, the majority of them took the form of a table or checklist to be filled out, containing a series of key questions or issues to consider. Although this format is highly adaptable across a broad range of scenarios, it is highly subjective and would likely result in variable, often arbitrary scoping outcomes depending on who is conducting the scoping.

The first area of consideration focused on stakeholder engagement, which is a key component of the HIA practice and is often introduced in the scoping step. The minimum elements and practice standards state that “a stakeholder engagement plan should be developed that establishes not only which stakeholders should be invited to participate in the process, but also the level of engagement to be solicited, and the methods that will be utilized to promote stakeholder participation throughout the HIA process” (6). While two of the tools included fairly detailed discussion and/or consideration of stakeholder engagement, including identifying key parties and engagement approaches, the rest either did not address stakeholders in the scoping step at all or provided limited consideration of the issue. For example, asking the question “who are the key stakeholders?” was posed without providing any additional information or process details. Such a limited approach would likely not result in a comprehensive stakeholder engagement process nor does it foster transparency or reproducibility within HIA scoping.

The next aspect of scoping included in the analysis was whether there is any provision for the selection of determinants, which has been identified as a key practice standard: “the range of health issues to be examined in the HIA should be clearly defined” (6). Out of the 10 scoping tools analyzed, only 3 provided clear guidance on identifying potential health issues, by providing either a list of health determinants that the user can review and select from or a series of example pathway diagrams to show impact linkages. Although other tools touched on the importance of identifying potential health determinants, they were limited to having the user respond to answer the question of “which health determinants will be included” or by leaving space for a pathway diagram to be created. This approach may work in situations where the person conducting the scoping has a strong background in HIA and environment–health interactions but would be very difficult for novice or even intermediate users to complete without additional guidance or specific process requirements.

Establishing a plan to carry out the HIA was the next factor considered in the analysis. Despite the fact that the entire objective of scoping is to create a plan or blueprint for the HIA, many of the tools provided only limited process guidance on doing so. In the minimum practice standards, it is stated that “a plan for conducting the HIA should be established” with a list of several factors to be considered in developing the plan, including impacts, boundaries, evidence sources, research methods, roles and responsibilities, and information dissemination (6). The majority of the tools provided a series of questions addressing key issues to be considered in conducting the HIA, including those listed in the minimum practice standards; however, many of the tools failed to address several of the important factors listed.

The next two sections included analysis of whether a systematic method of focusing on impacts of greatest significance was included in the scoping tool, and whether priority health issues were identified based on key factors. Both of these aspects of scoping are identified in the minimum practice standards, which discuss the need for systematic determination of potential health impacts and causal pathways, as well as determining priority issues to include in the HIA:

“The final scope should focus on those impacts with the greatest potential significance, with regards to factors including but not limited to magnitude, severity, certainty, stakeholder priorities, and equity. [Additionally] in identifying and evaluating priority health issues, practitioners should consider the expertise of health professionals, the experience of the affected communities, and the information needs of decision-makers” (6)

Based on this analysis, these two aspects represent the areas with the largest methodological gaps in HIA scoping. Out of the 10 tools considered, 6 of them provided no systematic process for focusing on impacts of greatest significance, and 4 of them noted the importance of prioritizing health determinants but provided no methodology for achieving this objective. As for prioritization of determinants using key factors, only one tool provided a process for using “impact” and “likelihood” to help inform priority; however, there was no clear decision-making process for determining the outcome based on these factors and therefore remains somewhat subjective and limiting in terms of reproducibility. Overall, the notion of using key factors for systematic prioritization of health determinants for inclusion in HIA is the largest methodological gap in HIA scoping.

With respect to identifying the roles and responsibilities of those involved in the HIA, all but one of the tools acknowledged this element as a key aspect of scoping. However, inclusion was typically limited to asking a question or series of questions pertaining to specific roles (i.e., project manager, expert, etc.), but in some cases more detail was required. For example, one of the tools included comprehensive terms of reference that needed to be signed off and included information on roles and responsibilities of team members. Although the level of effort dedicated to completing this aspect of scoping is likely variable, it is an important and underrated part of planning an HIA. When the roles and responsibilities are unclear, the expectations can become misaligned, which can result in future problems.

The final aspect of the analysis pertained to budget considerations. Although the minimum practice standards do not directly address budget in HIA scoping, lack of budgetary consideration is often stated as one of the major obstacles to conducting HIA. Specifically, budget restrictions can play a major role in scoping, since the decision to include or exclude certain determinants is often not solely based on the level of impact or concern but is based on the amount of funding and resources available for the HIA, which can be highly variable. Although many of the tools did acknowledge budgetary considerations, they were almost exclusively limited to a single question asking about the “budget and resources” available. No process or method of including the impact of budgetary factors in the selection of determinants as part of the scoping process was identified in any of the tools.

### Addressing Gaps: Development of a Systematic Scoping Method

With the volume of HIA guidance documents and tools that are available, creating another resource that duplicates efforts is not beneficial. Rather, addressing gaps in existing HIA scoping methodologies, as identified in the comparative analysis, can provide a way forward by building off of existing resources. For this reason, the methodology developed here focuses on the systematic characterization and prioritization of health determinants, addressing data gaps, and budget allocation. Although it may be used as a stand-alone tool in some instances, it can also be applied as a supplement to other guidance documents and HIA toolkits.

**Figure 3:**
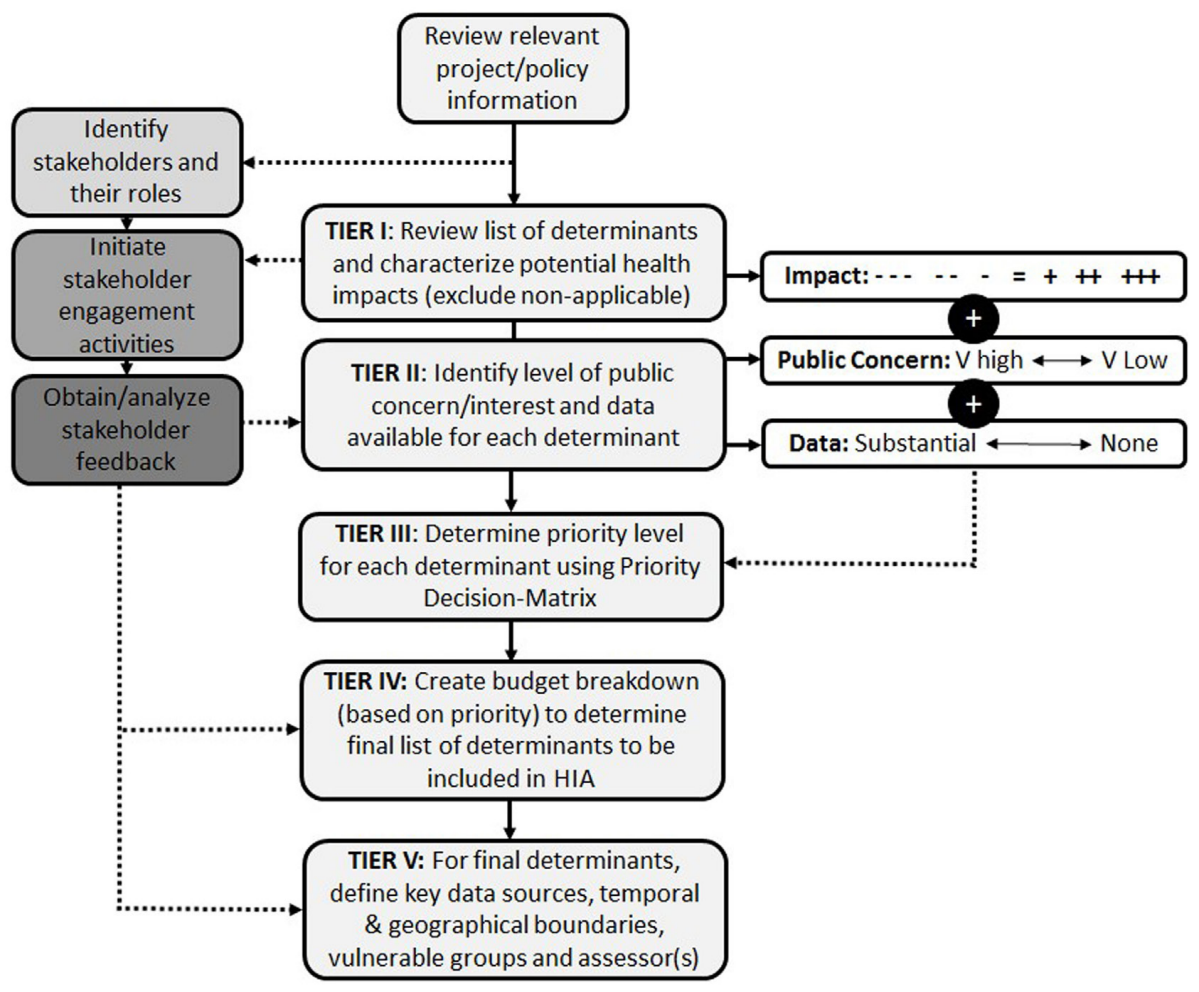
**HIA scoping tool overview: a systematic tiered approach to prioritizing health determinants for inclusion in the HIA**.

The initial concept of a systematic scoping tool was developed to mirror the tiered approach applied to systematic literature reviews. In a systematic literature review, screening of various pieces of information based on specific criteria promotes a transparent method of systematically weaning down a large volume of information to identify the most relevant and valuable aspects for inclusion (7). Applying this basic concept to an HIA scoping tool resulted in a tiered approach to prioritizing a large number of potential determinants and distilling them down to a justifiable priority list for inclusion in an HIA. This resulted in a tiered approach to systematically screening determinants for inclusion in HIA that ideally should be informed by a combination of scientific evidence and stakeholder input (Figure 3).

In order to enhance transparency, each aspect of the Systematic HIA Scoping Tool had to be clearly defined to promote consistent application by a variety of users and under differing scenarios. Specific factors were selected as key indicators for which determinants to include in an HIA. The most important factor was impact, which describes the potential for the proposed project/policy to positively or negatively affect human health. This was considered the primary factor in determining priority based on the premise that if there is no potential for impacting health, then there is no reason to include it in an HIA. Further, the degree of impact (i.e., minor vs. major) should dictate the relative importance of including that determinant in the assessment. The second factor included in determining priority was the level of public concern or interest. This factor was selected based on the fact that one of the pillars of HIA is democracy, and the involvement of the public and other stakeholders is a key component of the practice of HIA. In contrast to the first factor (impact), which is largely informed by scientific evidence and data, public concern/interest is informed by more subjective sources, such as media coverage, opposition, and contact with local communities. By ensuring that those determinants of importance to the public are included in the HIA, the process is aligned with the founding principles of the practice. For example, at times, the public is concerned about issues that are not those of most concern from an impact perspective. The final factor was data availability, which was a practical consideration for prioritizing and/or excluding certain health determinants. In cases where there is little to no data available for a particular determinant, this can provide a basis for exclusion or the requirement of additional studies to obtain the data. For each of these factors, separate definitions were developed to maintain as much consistency in the application of the methodology as possible; however, some level of interpretation, particularly in terms of the interdependence of different priority outcomes, is inevitable. For each health determinant, the tool requires that the following questions be answered in order to determine the priority level (Table 2).

**Table 2:** **Definitions for characterizing health determinants in Tier I and Tier II scoping**.

**Impact: what is the potential impact on human health?**	
N/A	*Not applicable*: the determinant is not applicable to the policy/project under consideration
+++	*Highly positive impact*: there is potential for a significant and long-term/permanent effect that could directly or indirectly improve health and wellness
++	*Moderately positive impact*: there is potential for a modest and short-term/temporary effect that could directly or indirectly improve health and wellness
+	*Slightly positive impact*: there is potential for a minimal and short-term/temporary effect that could directly or indirectly improve health and wellness
=	*Neutral impact*: although relevant, the effect is undetectable, even under worst-case scenarios, resulting in no impact to health and wellness
−	*Slightly negative impact*: there is potential for a minimal and short-term/temporary effect that could directly or indirectly diminish health and wellness
− −	*Moderately negative impact*: there is potential for a modest and short-term/temporary effect that could directly or indirectly diminish health and wellness
− − −	*Highly negative impact*: there is potential for a significant and long-term/permanent effect that could directly or indirectly diminish health and wellness
**Public concern: what is the level of public concern/interest?**	
VH	*Very high*: extreme concern/interest over proposal and/or impacts with lots of media coverage, strong opposition groups, including protests, and excess public feedback/correspondence (online or in person). Very high (top 10%) priority ranking in majority of stakeholder engagement feedback
H	*High*: high level of concern/interest over proposal and/or impacts with some media coverage, moderate opposition/concern, and consistent public feedback/correspondence (online or in person). High priority (top 10–20%) ranking in majority of stakeholder engagement feedback
M	*Medium*: moderate level of concern/interest over proposal and/or impacts with sparse media coverage, mild opposition/concern, and some public feedback/correspondence (online or in person). Medium priority ranking (top 20–50%) in majority of stakeholder engagement feedback
L	*Low*: low level of concern/interest over proposal and/or impacts with little to no media coverage, minimal opposition/a few concerned individuals, and limited public feedback/correspondence (online or in person). Low priority ranking (bottom 20%) in majority of stakeholder engagement feedback
VL	*Very low*: very low level of concern/interest over proposal and/or impacts with no media coverage, no known opposition/concern, and no public feedback/correspondence (online or in person). Very low to no priority ranking (bottom 10% or not included) in majority of stakeholder engagement feedback
**Data availability: what is the availability of data?**	
A	*Substantial*: there is a high volume of relevant data readily available, all of which is at an appropriate scale (i.e., local/regional/global) with minimal data gaps
B	*Partial*: there is a moderate to low volume of relevant data readily available, some of which is at an appropriate scale (i.e., local/regional/global), with some key data gaps. Some additional data may be required to be collected/obtained, if possible
C	*Very limited*: there is a negligible volume of relevant data available, almost none of which is at an appropriate scale (i.e., local/regional/global), with several important data gaps. Large amounts of additional data may be required to be collected/obtained, if possible
D	*None*: there is no quantitative or qualitative data available. It is not possible to collect/obtain additional data

Collectively, the characterization of these three factors provides a foundation for determining prioritization of all applicable health determinants. In some cases, there may be limited to no stakeholder input available at the time scoping is conducted. In this case, reliance on professional judgment and case studies from similar HIAs may be necessary, although not ideal. Further, where a health determinant is not applicable to the policy or project under consideration, it can be identified as “N/A” in Tier I and excluded from the assessment.

### Priority Decision-Matrix

**Figure 4:**
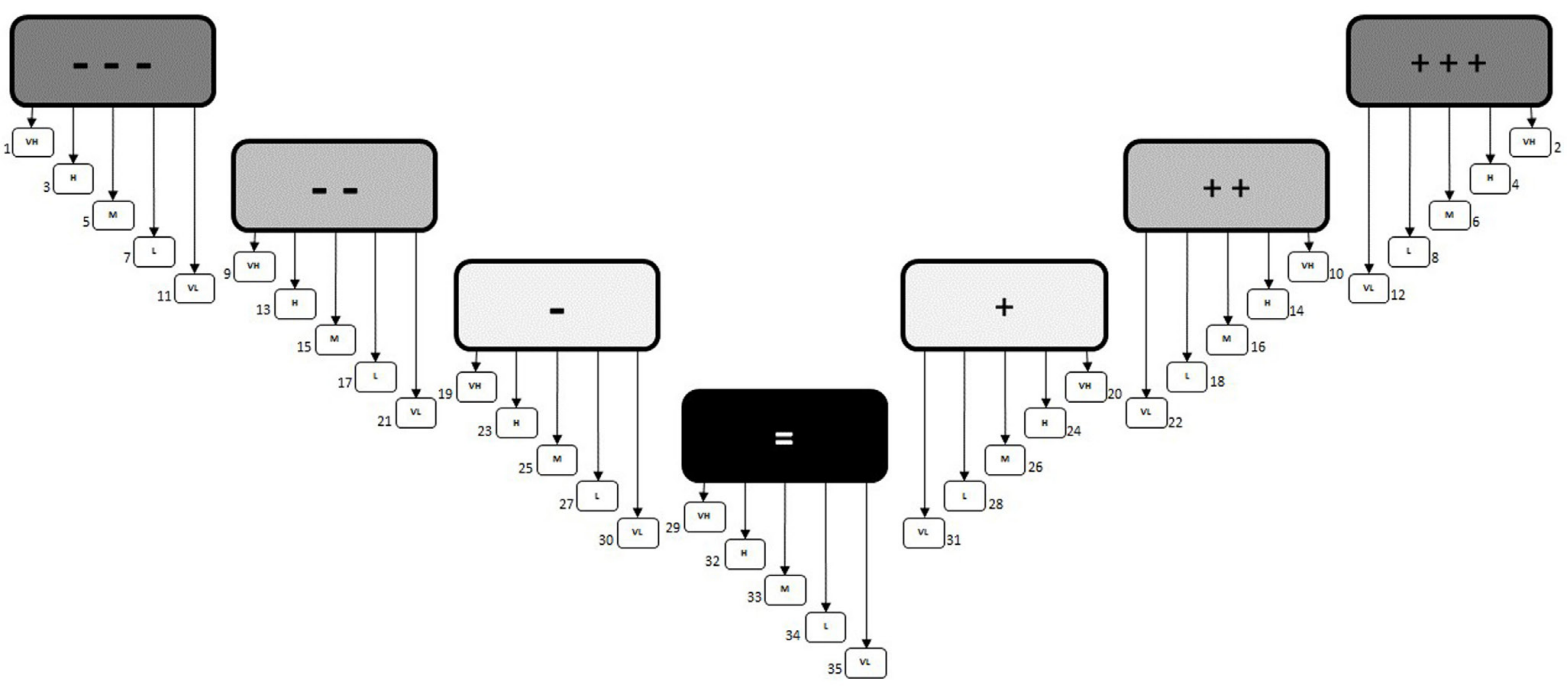
**Priority Decision-Matrix (version 1.0)**. The initial decision matrix that was based on having a different priority number assigned to each unique outcome.

In order to facilitate a transparent, reproducible, and widely applicable method of prioritizing health determinants, a Priority Decision-Matrix was created. The first version of this matrix was based on the concept that based on a combination of “impact” (large boxes: − − − to +++) and “public concern/interest” [small boxes: VH (very high) to VL (very low)], a priority order could be identified for each unique outcome; resulting in 35 distinct outcomes, each assigned a different priority order accounting for both positive and negative impacts (Figure 4).

**Figure 5:**
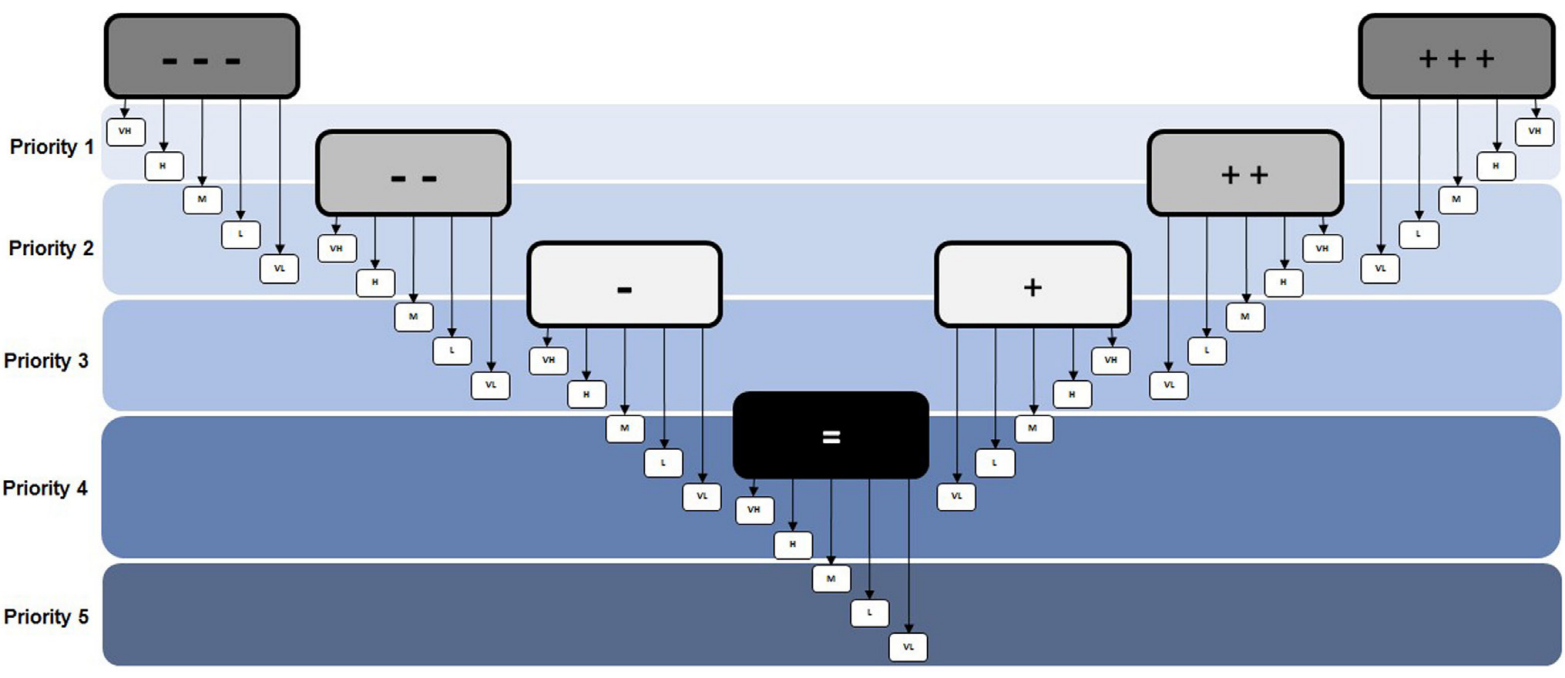
**Priority Decision-Matrix (version 2.0)**. The revised decision matrix that was based on groupings or bands to determine priority rather than individual outcomes.

However, the problem with this approach was realized following some initial testing where the priority order could not be adequately justified because the outcomes were not unique enough from each other. In other words, having 35 distinct priority outcomes (#1 to #35) was too specific, making it too difficult to account for the, often minor, differences that would distinguish one result from another. To address this issue, it was determined that fewer priority outcomes were needed. That way, there would be distinct and unique aspects of each outcome that justify the prioritization without reducing the number of options for characterization. Therefore, it was concluded that the priority was better suited to groupings or “bands” (Figure 5). This way, several potential outcomes were identified within the same priority group and could be fully justified when compared to other outcomes. Additionally, although negative outcomes tend to receive more focus in practice, it was considered important to categorize positive impacts as having the same relative level of importance as negative ones, since evaluating both impacts and benefits is a cornerstone of HIA.

### Addressing Data Gaps

**Figure 6:**
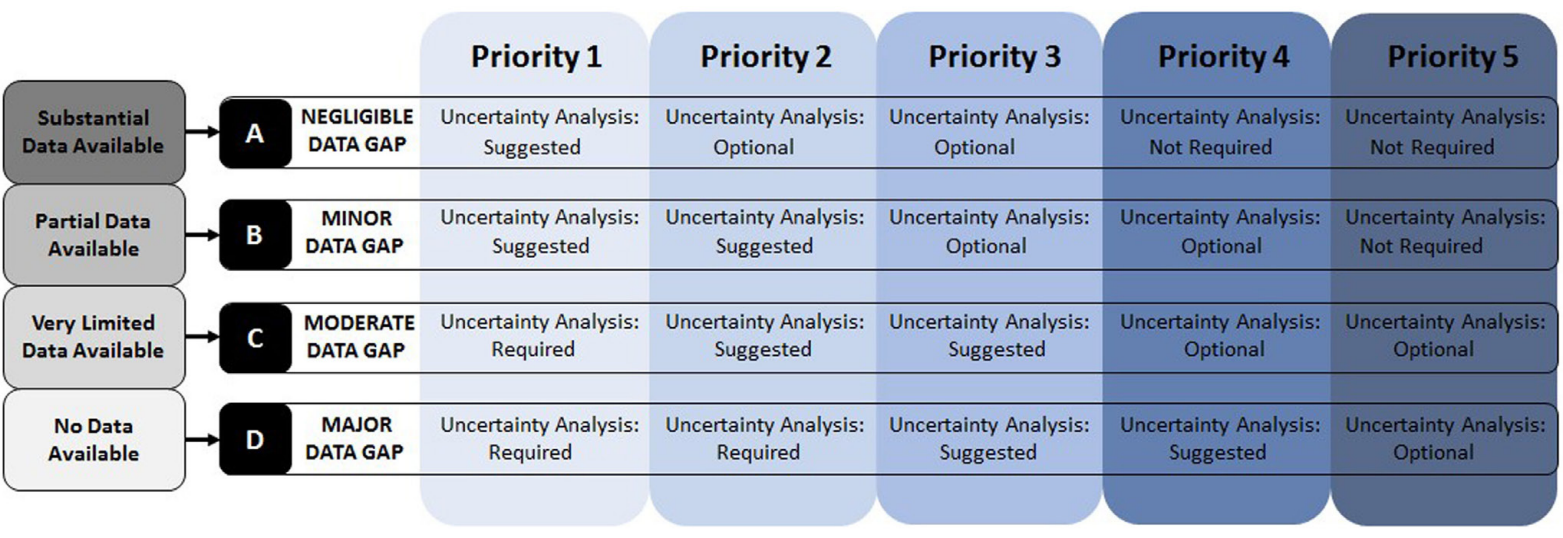
**Data gap identification and uncertainty analysis**. Based on data availability, gaps are identified, and uncertainty analysis guidance is provided.

The third factor considered in determining priority is the availability of data. This aspect had to be included in a unique way since the amount or quality of data is important in that it defines the level of assessment that can be done, but the absence or presence of data alone should not govern the priority ranking of potentially harmful (or beneficial) effects. Therefore, in order to adequately address this issue, a sub-ranking was included to identify the data available for evaluation (A: substantial; B: partial; C: very limited; D: none). This sub-ranking is then included in the overall priority listing without changing the initial prioritization determined by impact and public concern. The value of this additional ranking is that it assists in further scoping of the HIA by identifying any major or minor data gaps and providing guidance on conducting uncertainty analysis (Figure 6).

Based on the combination of priority order (1–5) and data availability (A–D), the data gap classification and requirements for uncertainty analysis are provided. This ensures that any high priority determinants that are lacking in data are considered in the HIA in a transparent manner. The following definitions guide the requirements set out in Figure 6.

#### Uncertainty Analysis

Uncertainty analysis must include a detailed discussion of the implications of having a lack of data for a specific determinant, and whether it implicates the conclusions of the HIA as a whole. There are different requirements for the uncertainty analysis depending on the determinant priority level and availability of data:

- *Required*: an extensive analysis of the limitations and uncertainty associated with the importance/priority and data availability, including discussion of the implications for the overall HIA findings, is required.
- *Suggested*: a moderate analysis of the limitations and uncertainty associated with the importance/priority and data availability, including discussion of the implications for the overall HIA findings, is suggested if budget and resources are available. If not, this must be explicitly stated in the HIA as a limitation.
- *Optional*: a limited-scope analysis of the limitations and uncertainty associated with the importance/priority and data availability, including discussion of the implications for the overall HIA findings, is optional. No limitations statement is required.
- *Not required*: no uncertainty analysis is required.

Following prioritization of determinants, including evaluation of the potential health impacts, public interest/concern, and data availability, the tool includes a step for consideration of other factors that may impact prioritization. These factors include things that would require deviation from the existing priority order, such as equity issues, other stakeholder concerns, uncertainty around impacts, and others. The prioritization of determinants can be changed to reflect these other factors as long as some justification or explanation is provided to maintain transparency within the scoping process.

### Budget and Resource Allocation

After the final priority order has been determined, budget allocation can be completed. This tool is not intended to provide an overall costing for HIA. Instead, it takes an HIA budget and requires the user to allocate funds for the assessment of specific determinants. This process will ensure transparent allocation of resources to those determinants that have the highest priority. Additionally, if there are any key determinants that cannot be assessed due to budget restrictions, this tool provides a clear and consistent way of acknowledging this fact as a limitation of the HIA. Conversely, if too few determinants can be evaluated with existing funds, then it can provide a basis for requesting additional resources.

The first step in allocating budget is to determine how much is available for the assessment step. It is important to note that this amount should not include other costs associated with conducting the HIA, such as project management, administrative tasks, report writing, monitoring and evaluation, stakeholder engagement activities, etc. To determine the overall cost of an HIA, there are existing tools that can be used (18). Once the “assessment only” budget has been determined, the amounts required to assess each of the determinants should be applied, moving down the list in order of priority (highest to lowest). The concept behind this strategy is that applying budget to the assessment of determinants allows for a transparent method of inclusion in the HIA, while ensuring that the highest priority issues are considered. Allocating budget, or expected costs, to each of the determinants should continue until either the full list of determinants is included in the HIA or the budget runs out. In cases where there is insufficient budgetary allocation to include all priority determinants, a decision must be made about whether to request additional funding or acknowledge the potential limitation of excluding certain determinants from the assessment. When deciding which determinants to include/exclude, the process should also consider opportunity costs associated with not evaluating the potential impacts of certain determinants.

### Automation of the Systematic HIA Scoping Tool

The HIA scoping tool analysis found that in addition to specific gaps in methodology, there was a lack of automated tools to promote efficiency of the scoping process. Therefore, the Systematic HIA Scoping Tool was automated to increase efficiency and utility. This automation was considered vital since lack of time and budget to carry out HIA is one of the main issues within the practice (4, 19). Programing the tool in Microsoft Excel was considered essential due to the ease of use and familiarity of the program to a wide variety of potential users. Additionally, Excel provided the necessary platform to build the tool in such a way that it simplified functionality by allowing the priority decision-matrix to be built into the spreadsheet; thereby facilitating an otherwise complex and onerous process.

The foundation of the tool is a comprehensive list of over 70 determinants of health that were compiled from numerous well-known HIA resources (3, 8, 10, 20–22), resulting in the most comprehensive list of determinants included in any of the HIA scoping tools reviewed. After the list of health determinants was finalized, the Systematic HIA Scoping Tool was built into excel in a table format where users could provide inputs from drop-down menus, and the tool would automatically provide a priority order for all relevant determinants, based on the decision matrix outputs. Once all of the required fields have been filled, a “sort” button reorganizes the health determinants list in order of priority to easily summarize and group the most relevant issues. The priority order is based on a combination of the inputs for “impact on health,” “public concern/interest,” and “data availability.” In special cases, users are able to deviate from the identified priority order by manually reassigning priority but must provide a justification to do so. For example, if a specific determinant is listed as a priority #3, but the local public health agency has it ranked as a top priority for their region, it can be reassigned priority #1 or #2 providing this explanation as a rationale for the adjustment.

**Figure 7:**
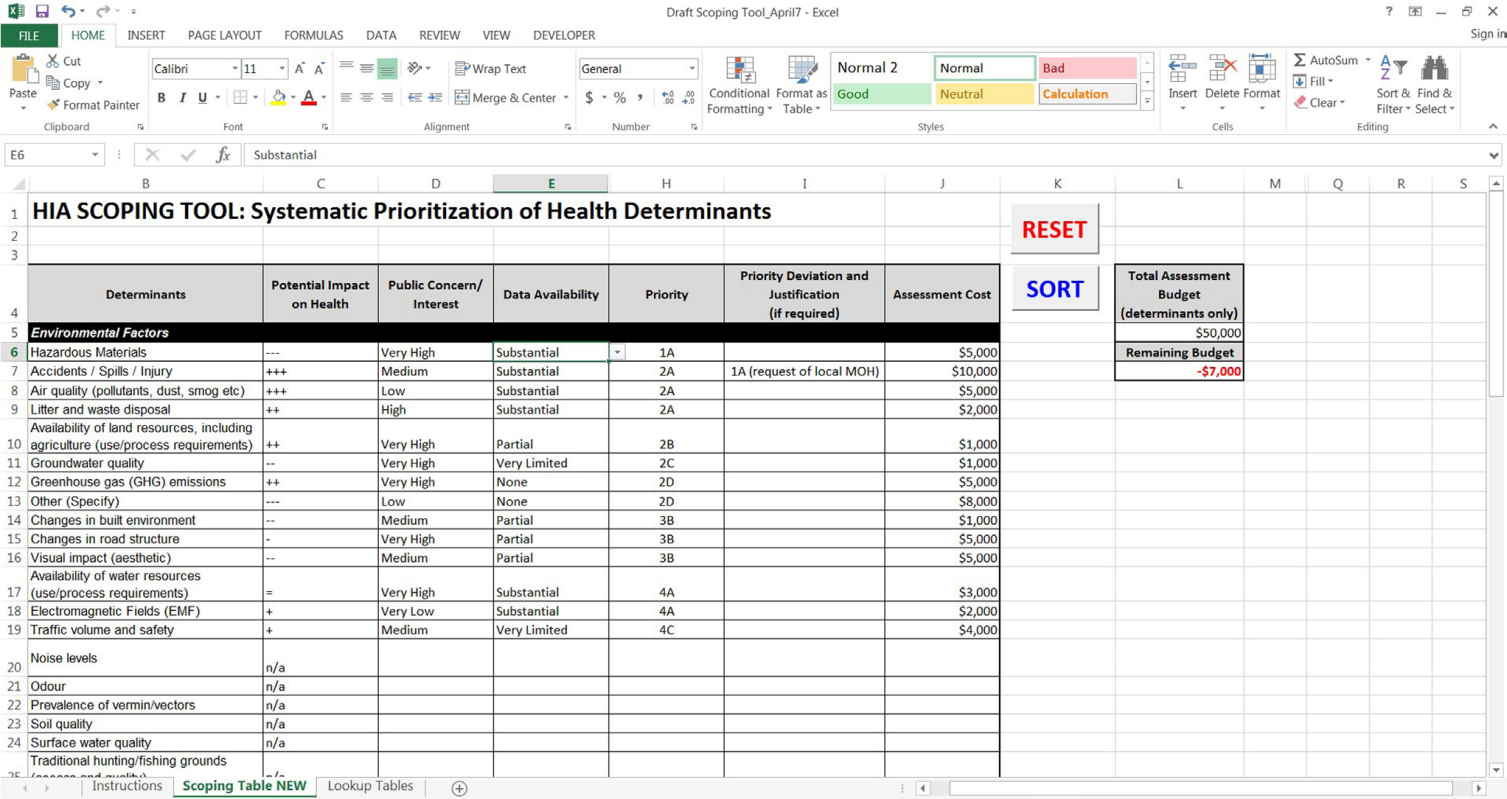
**Systematic HIA Scoping Tool: example (automated in Microsoft Excel)**.

Once the final priority order has been determined, the user can then input their total assessment budget and allocate costs for assessing each of the determinants. As the costs are assigned, the remaining budget will automatically be calculated, and if the budget is exceeded, the value turns red to alert the user. This feature is a key aspect of the Systematic HIA Scoping Tool because it ensures that the highest priority determinants are included for assessment and provides a basis for exclusion of lower priority determinants, especially when budget and resources are limited (Figure 7). To request an automated version of the Systematic HIA Scoping Tool, please contact the corresponding author.

In the example provided in Figure 7, a theoretical characterization of environmental factors shows how the tool functions to provide a priority list based on impact and public concern (#1–5) as well as data gaps (A–D). In this example, the determinants have been sorted in order of priority, and in one case, the priority order was deviated from with justification (i.e., “at the request of the local medical officer of health”). The total assessment budget was then entered, and the assessment costs for each determinant were listed. In this example, the cost of assessing all of the determinants exceeds the budget (−$7,000), which is indicated in red as the “remaining budget.” In such cases, when the determinant list exceeds the allocated budget, the HIA team can either secure additional funds to include all determinants or focus available resources on the higher priority determinants and provide a justification for exclusion of lower priority determinants. In addition, if the HIA is being conducted by public servants, it may be preferable to allocate hours rather than costing. In this case, the “assessment cost” column can be used to enter hours for assessing each determinant rather than monetary values to ensure there is enough time to complete the HIA.

### Finalizing the HIA Scope of Work

The final step of the scoping process consists of defining boundaries for those determinants that have been included for assessment. Once all factors have been considered and the budgetary constraints have been applied in the automated tool, the final list of priority health determinants to be carried forward in the HIA should be listed in the following table along with all required information needed to conduct the assessment step (Table 3).

**Table 3:** **Scope of work for final priority health determinants**.

Final health determinants (priority #)	Key data sources	Temporal boundaries	Geographical boundaries	Vulnerable population	Key contact and role
Example: noise (1A)	Noise modeling project report; local noise monitoring data	Short-term: construction phase	Local study area (5 km)	Children	J. Smith (modeling and impact assessment)
Example: employment (2B)	Local census data; project employment information	Long-term: construction and operation phases (30 years)	Regional study area (100 km)	Unemployed	R. Johnson (review and assessment)
Etc.…					

It is important to note that this Systematic HIA Scoping Tool is intended for users who have experience in HIA and have sufficient knowledge around environment and health interactions. This allows decisions to be made around potential impacts, which are largely based on early limited information and can be better informed during the assessment step, with an informed awareness of the potential for positive or negative outcomes resulting from proposed project or policy initiatives.

## Discussion

The literature review and comparative analysis of existing HIA scoping tools found that there was a clear lack of systematic methods and processes for prioritization and selection of health determinants. Therefore, a novel methodology was developed based on application of a priority decision-matrix that characterizes key factors (i.e., impact, public concern/interest, data availability) to inform an assessment hierarchy. Combining this approach with consideration of data gaps, uncertainty analysis and budget allocation provide a more transparent, systematic, and reproducible approach to HIA scoping.

### Rationale for Systematic Prioritization

The foundation of the Systematic HIA Scoping Tool is the priority decision-matrix, which uses key factors to inform a priority order for assessment of health determinants. The main factors that make up the basis of the decision matrix are (i) impact, (ii) public concern/interest, and (iii) data availability. These factors were selected because they align with the pillars of HIA as well as the Minimum Elements and Practice Standards. The four main pillars of HIA are democracy, equity, sustainable development, and ethical use of evidence (23). Democracy is defined as “emphasizing the right of people to participate in a transparent process for the formulation, implementation and evaluation of policies that affect their life, both directly and through the elected political decision makers” (23). This concept was considered in the Systematic HIA Scoping Tool by including public input into the prioritization of health determinants. By involving these stakeholders in the scoping process in a way that makes a real impact to the outcome of the HIA, the tool remains consistent with the core values upon which HIA is based. Additional discussion around consideration of stakeholder input is provided below.

**Figure 8:**
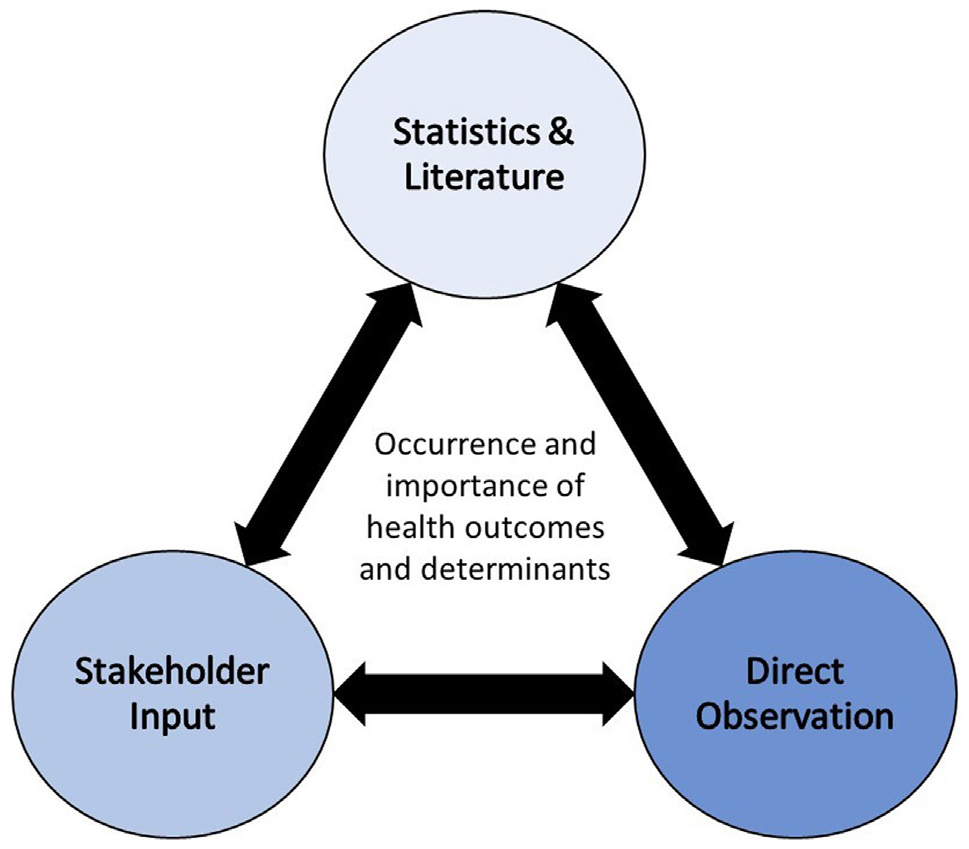
**Methodological triangulation to determine the occurrence and importance of health outcomes and determinants (24)**.

The other factor that was key to determining a priority order for health determinants was impact: “Health impacts are the overall effects, direct or indirect, of a policy, strategy, program or project on the health of a population” (23). The impact of a specific determinant is dependent on the nature of the project, policy, or program being evaluated. Therefore, a certain level of detailed information should be available for review prior to making the decision regarding potential impacts. Additionally, characterizing impacts of specific activities on various determinants of health can be a complex process that should ideally be carried out by individuals with expertise in the field of HIA and having a strong understanding of human–environment interactions. Defining the occurrence and importance of impacts associated with health determinants should be largely informed by established science, as published in the primary literature, and/or direct observation, in combination with stakeholder input [(24); Figure 8]. When deciding on a hierarchy of evidence to inform scoping, the strongest sources are from the primary literature (e.g., meta-analyses, reviews, studies), followed by evidence provided by key informants and stakeholders (19). Based on this assumption, the Systematic HIA Scoping Tool relied first on impact evidence, informed by published literature and scientific fact, and then considered stakeholder/public input as secondary when determining priority.

In addition to impact and public input, the Systematic HIA Scoping Tool incorporates the “ethical use of evidence” pillar by taking into consideration the quality and availability of data. The WHO (23) states that “the use of quantitative and qualitative evidence has to be rigorous, and based on different scientific disciplines and methodologies to get as comprehensive assessment as possible of the expected impacts.” By conducting a preliminary evaluation of the data gaps associated with each relevant health determinant, the tool promotes a transparent approach to the use of evidence in HIA. It also allows for upfront acknowledgment and communication of the limitations and possible uncertainties associated with the assessment.

### Stakeholder Engagement in HIA Scoping

One of the least consistent areas of HIA practice is stakeholder engagement. Although it is often identified as a key component of HIA, the level of rigor in stakeholder identification, engagement, and involvement in the process is highly variable (25). Often, the first opportunity for stakeholders to get involved is during the scoping step, where they can inform decisions about the plan for the HIA. The Minimum Elements and Practice Standards states that:

“Meaningful and inclusive stakeholder (e.g., affected community, public agency, decision-maker) participation in each step of the HIA supports HIA quality and effectiveness. Each HIA should have a specific engagement and participation approach that utilizes participatory or deliberative methods suitable to the needs of stakeholders and context” (6).

As part of the review and analysis of existing HIA scoping tools, identification of a stakeholder engagement approach was considered a key component of a robust scoping methodology. Although several of the HIA scoping tools acknowledged the need for some sort of stakeholder engagement and some provided examples of typical stakeholders and common engagement activities, they were not consistent or specific in their approach or application.

Recently, a collaboration between the Center for Community Health and Evaluation and Health Impact Partners resulted in a detailed report entitled “Community Participation in Health Impact Assessments: A National Evaluation,” examining the varying levels of community participation in HIA in the United States (26). The results of the national evaluation found that one-third of respondents ranked the level of community participation on the low end of the spectrum (either inform or consult) without providing details on how their feedback would be incorporated into the HIA process (26). Additionally, the evaluation looked at community participation methods and compared utility with effectiveness. Although obtaining feedback/comments on a draft HIA was the most popular method of participation, followed by public meetings and then inclusion on a steering committee, the most effective method was key informant interviews. Despite the level of variability in community participation, of the 47 HIAs surveyed, 84% reported that it had a “positive” or “very positive” impact on the success of the HIA (26).

**Figure 9:**
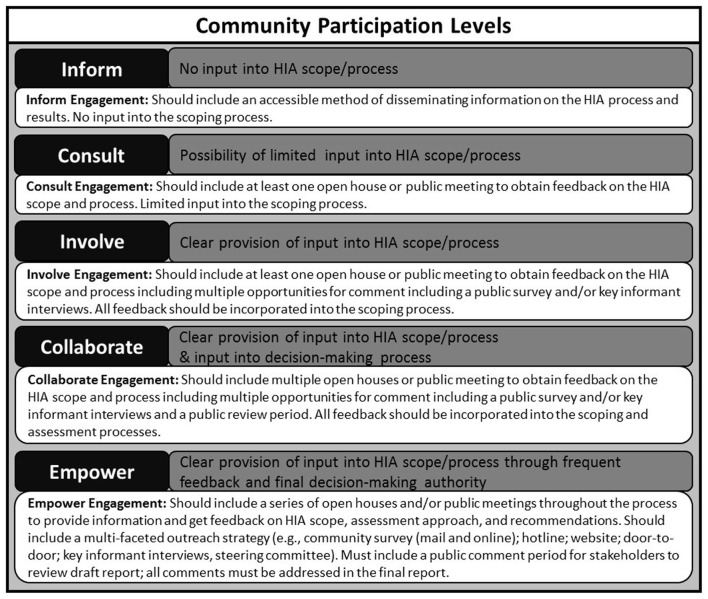
**Levels of stakeholder engagement in HIA scoping. Community participation levels adapted from Ref. (26)**.

Overall, they identified a range of “community participation levels” including inform, consult, involve, collaborate, and empower (26). These participation levels were adapted and used to provide some preliminary guidance on incorporating stakeholder feedback into the HIA scoping process (Figure 9). At a minimum, it is vital that HIA practitioners acknowledge the importance of community input and are upfront about the level of influence that various stakeholders have on the process.

### Limitations and Future Research

Although the Systematic HIA Scoping Tool was developed in order to address current gaps in the HIA scoping methodologies, including a lack of transparency and reproducibility, there are some limitations to this approach. First, the literature review and comparative analysis focused on HIA scoping tools rather than general guidance documents, which omitted a large volume of work. There may be some information and process suggestions that would enhance HIA scoping; however, the variability in the application of general guidance is typically high. For this reason, the authors focused on tools that have been specifically developed to assist with the consistent application of a scoping process.

Second, the Systematic HIA Scoping Tool was developed by the authors and thus has inherent assumptions and personal judgments. As in any development process, certain decisions had to be made about how to proceed. This is especially true for the priority decision-matrix that was developed to provide a consistent, transparent, and systematic approach to selecting priority health determinants for inclusion in HIA. The specific factors that were selected and the definitions that were developed are intended to promote objectivity in the process; however, with different users applying the tool to various scenarios, complete objectivity is not possible. Despite this reality, the authors agree that this approach does take a step in the right direction in terms of creating more consistent methods in HIA to ensure that the practice is robust, clear, and defensible.

In order to determine the efficacy of this type of scoping approach, the tool should be used by HIA practitioners and applied to a wide range of potential scenarios. In order to properly test the Systematic HIA Scoping Tool, it requires specific information on a project, policy, or program and public/community input; therefore, it is not conducive to testing using theoretical case studies. Areas of future research that should be pursued are to test the tool on large- and small-scale projects in a variety of sectors, various policy initiatives, and a range of simple to complex program proposals in different jurisdictions. In some cases, the tool may benefit from enhancement based on feedback on various aspects including, effectiveness, ease of use, comprehensiveness of content, agreement with existing scoping processes, and other key aspects of the tool content and functionality.

Overall, the Systematic HIA Scoping Tool was developed based on the identification of methodological gaps in existing scoping practices, and it provides practitioners with a more transparent and consistent method of identifying priority health determinants for inclusion in HIA. Future work in the area of HIA methodology development is vital to the ongoing success of the practice and utilization of HIA as a reliable decision-making tool.

To request an automated version of the Systematic HIA Scoping Tool, please contact the corresponding author at lindsay.c.mccallum@gmail.com.

## Author Contributions

LM, CO, and IS contributed to the iterative process associated with development of the HIA Scoping Tool, including critical review and revisions. All authors contributed to the manuscript and approved the final version to be published.

## Conflict of Interest Statement

The authors declare that the research was conducted in the absence of any commercial or financial relationships that could be construed as a potential conflict of interest.
